# Fundamentals of Membrane Lipid Replacement: A Natural Medicine Approach to Repairing Cellular Membranes and Reducing Fatigue, Pain, and Other Symptoms While Restoring Function in Chronic Illnesses and Aging

**DOI:** 10.3390/membranes11120944

**Published:** 2021-11-29

**Authors:** Garth L. Nicolson, Gonzalo Ferreira de Mattos, Michael Ash, Robert Settineri, Pablo V. Escribá

**Affiliations:** 1 Department of Molecular Pathology, The Institute for Molecular Medicine, Huntington Beach, CA 92647, USA; 2 Laboratory of Ion Channels, Biological Membranes and Cell Signaling, Department of Biophysics, Facultad de Medicina, Universidad de la República, Montevideo 11600, Uruguay; ferreira@fmed.edu.uy; 3 Clinical Education, Newton Abbot, Devon TQ12 4SG, UK; michaelash@clinicaleducation.org; 4 Sierra Productions Research, Mission Viejo, CA 92612, USA; gnicolson3@immed.org; 5 Laboratory of Molecular Cell Biomedicine, University of the Balearic Islands, 07122 Palma de Mallorca, Spain; pablo.escriba@uib.es

**Keywords:** membrane phospholipids, membrane structure, lipid transport, lipid oxidation, lipid exchange, mitochondrial function, fatigue, pain, chronic diseases

## Abstract

Membrane Lipid Replacement (MLR) uses natural membrane lipid supplements to safely replace damaged, oxidized lipids in membranes in order to restore membrane function, decrease symptoms and improve health. Oral MLR supplements contain mixtures of cell membrane glycerolphospholipids, fatty acids, and other lipids, and can be used to replace and remove damaged cellular and intracellular membrane lipids. Membrane injury, caused mainly by oxidative damage, occurs in essentially all chronic and acute medical conditions, including cancer and degenerative diseases, and in normal processes, such as aging and development. After ingestion, the protected MLR glycerolphospholipids and other lipids are dispersed, absorbed, and internalized in the small intestines, where they can be partitioned into circulating lipoproteins, globules, liposomes, micelles, membranes, and other carriers and transported in the lymphatics and blood circulation to tissues and cellular sites where they are taken in by cells and partitioned into various cellular membranes. Once inside cells, the glycerolphospholipids and other lipids are transferred to various intracellular membranes by lipid carriers, globules, liposomes, chylomicrons, or by direct membrane–membrane interactions. The entire process appears to be driven by ‘bulk flow’ or mass action principles, where surplus concentrations of replacement lipids can stimulate the natural exchange and removal of damaged membrane lipids while the replacement lipids undergo further enzymatic alterations. Clinical studies have demonstrated the advantages of MLR in restoring membrane and organelle function and reducing fatigue, pain, and other symptoms in chronic illness and aging patients.

## 1. Introduction: Why Membrane Lipid Replacement (MLR)

Membrane lipids are essential to life and vital to cellular health [1,2,3,4,5]. They provide cells with: (i) a matrix for all plasma and intracellular membranes, (ii) separation of chemical and enzymatic reactions into distinct cellular compartments; (iii) bioactive molecules that are part of signal transduction and molecular recognition pathways; (iv) energy storage reservoirs; and (v) molecules that interact with other membrane constituents, such as proteins and glycoproteins [1,2,4]. The interactions of membrane lipids with membrane proteins and glycoproteins is an absolute requirement for the formation, structure and function of cellular membranes and thus for life itself [1,2,3,4,5,6,7,8,9,10]. In addition, peripheral signaling proteins can translocate between the plasma membrane and cytoplasmatic aqueous or particulate (membrane) fractions. At the level of the lipid bilayer or membrane matrix, they propagate intracellular signals only if they exert productive interactions with transmembrane receptors in specific membrane nanodomains or submicrodomains. Such signals modulated by lipids can be replaced by pharma/nutraceutical interventions [11,12]. For these reasons, lipid membranes and other lipid structures constitute an essential element in a cell’s physiology, and lipid alterations within cells are likely to be involved in human disease. Thus, lipid normalization could be an approach to counteract certain aspects of pathological states.

During development, aging, and especially in chronic medical conditions and essentially every acute illness, cellular membranes are damaged, in part due to the excess production of free radical oxidants [13,14,15,16]. Free radical reactive oxygen species (ROS), such as superoxide anion radicals, hydroxyl radicals, and hydrogen peroxide, and reactive nitrogen species (RNS), such as peroxynitrite anion, cause most of the chronic damage to cellular membranes [13,14,17,18,19,20,21,22,23]. Cellular anti-oxidants usually neutralize excess free radical oxidants, but in pathologic conditions the concentrations of free radical oxidants are in such excess that normal anti-oxidants levels are ineffective and free radical oxidant damage occurs [17,23,24,25,26]. Membrane glycerolphospholipids (GPL) and their unsaturated fatty acids (FA) are especially sensitive to oxidative damage by ROS and RNS [1,17,25,26,27] and they must be enzymatically repaired or replaced in order to maintain membrane structure and function [1,2,3,4,7,9,12].

Under pathological conditions and normal aging, the use of Membrane Lipid Replacement (MLR), the natural replacement of membrane glycerolphospholipids (GPL) and other lipids mainly by oral dietary supplementation, can supply enough GPL and other lipids to repair damaged lipids and return cellular membranes to normal function [1,2,3,4]. Thus, membrane lipids can be maintained at optimal levels by complementing regular dietary membrane lipid sources with MLR, and this should provide enough replacement molecules for damaged cellular membrane lipids [1,2,3,4,5,6]. Membrane lipids and their derivatives can also be used therapeutically to treat various conditions and diseases [1,2,3,4,5,12].

Oral GPLs and their unsaturated fatty acids (FA) are quickly and efficiently absorbed, mainly in the upper small intestine, within hours of ingestion [1,2,3,28,29]. There are multiple mechanisms for absorption of orally ingested GPL. The ingested phospholipids can be degraded into their constituent parts and their component parts and separately absorbed; they can be taken in as intact molecules without degradation, or they can be absorbed in bulk in the form of small lipid micelles, liposomes, and globules [1,2,30,31]. When present in great excess in the gastrointestinal system during oral MLR supplementation, most membrane phospholipids are absorbed in undegraded bulk forms [30,31]. The intake process appears to be driven by a mass action or ‘bulk flow’ process [32]. Thus, when in large excess, intact MLR phospholipids have an advantage in being able to reach their final destinations at high concentrations by bulk flow without significant degradation or enzymatic alterations [1,2,32]. Once inserted at their ultimate cellular membrane and intracellular sites, the GPL can be further enzymatically modified, such as by substitution or modification of their FA side chains or head groups, in order to reflect the specific compositional and functional needs at their membrane destinations. At the cellular level the process of intracellular transport to organelles has been followed using spermatozoa. For example, human sperm cells have been incubated with nanometer sized GPL micelles loaded with some fluorescent-GPL molecules to follow their progress [33]. Fluorescence was first found at the sperm surface, and this was accompanied by an increase in plasma membrane surface area. Next, fluorescence could be found in the sperm intracellular membranes as well as in mitochondria. The presence of MLR molecules in the sperm mitochondria correlated with increases in sperm motility and resistance to oxidative stress [33].

## 2. Biological Membranes and GPL

GPL and other lipids are essential for biomembrane structure, properties, and function, and they form the matrix for all cellular membranes [1,2,8,9,34]. Along with GPL head-group, the chain length and saturation of attached FA help determine GPL membrane packing and membrane fluidity [8,9,34,35,36]. Unsaturated FA, such as oleic acid and linoleic acid, confer a high degree of conformational flexibility to GPL. The unsaturated FA hydrocarbon chains cause GPL to occupy a slightly wedge-shaped space in the hydrophobic interior of membranes, resulting in looser packing and a more fluid membrane [5,8,9,35,36]. For example, oleic acid provides special biophysical properties to membranes that are involved in the interactions of amphitropic peripheral signaling proteins that control important physiological functions, such as blood pressure [36]. In contrast, saturated FA, such as stearic acid and palmitic acid, confer membrane rigidity, and this results in a less fluid or more organized membrane structure [9,35,36]. Different plasma and intracellular membranes display GPL compositional differences that are characteristic of their origins [8,9,34,35].

The actions of various agents and drugs can affect the compositions of plasma membranes and in the process change their functional properties. For example, certain anti-platelet drugs, such as ticagrelor, a membrane P2Y12 antagonist, and its active metabolite significantly increased platelet cholesterol and PC with short FA acyl chains, suggesting platelet membrane rigidification and modification of lipid–receptor interactions involved in ADP-induced platelet activation [37].

There are also differences between the compositions of lipids on each side of the lipid bilayer of a given biomembrane. Sphingolipids, such as gangliosides, are distributed asymmetrically at much higher concentrations in the outer leaflets of plasma membranes. Neutral GPL, such as phosphatidylcholine (PC), are also found preferentially on the outer leaflets of plasma membranes, whereas anionic phospholipids, such as phosphatidylserine (PS) and phosphatidylinositol (PI), are more concentrated on the plasma membrane inner leaflet [8,9,34,35,38,39]. The asymmetric and cooperative distributions of lipids in inner and outer membrane leaflets, as well as in the plane of the membrane, are important in determining membrane physical properties, including membrane deformation, curvature, compression and expansion, as well as their functional interactions with membrane proteins [34,35,38,39,40,41]. These concepts have been incorporated in descriptions of membrane structure, as in the Fluid–Mosaic Model of cell membrane structure [42,43].

As described above, an important characteristic of plasma membranes is the association of lipids (and under certain circumstances, proteins) into functional membrane domains, such as lipid rafts [44,45]. This is due, in part, to lateral molecular interactions and lipid domain formation, especially between PC and phosphatidylethanolamine (PE), along with sphingomyelins and cholesterol [38,40,41,44,45]. Under physiological conditions, membrane lipids are present in various fluid, semi-solid, and solid phases that are organized into membrane domains that are characterized by different lipid compositions, spatial arrangements, and rates of lipid rotational and lateral movements [35,41,43,44,45,46]. The different lipid phases and domains in various membranes have profound functional significance in determining membrane properties, such as cell signaling and other important membrane characteristics [40,43,44,45].

Although cell membranes form functional ionic barriers, membrane ion channels and transporters function as conductive pathways for ions or water across such hydrophobic barriers, and biomembranes can thus behave as resistance-capacitor circuits with particular time and spatial characteristics that regulate trans-membrane voltage kinetics. These properties are critically important in membrane signaling and energy production [47,48]. The FA in membrane GPL that form most of the membrane hydrophobic barrier are present with a variety of FA chain lengths and saturation states. Thus, FA are important components of dietary MLR supplements, and among the most important FA for humans are unsaturated FA, such as oleic acid (18:1Δ9 or 18:1[n-9]), linoleic acid (18:2Δ9,12 or 18:2[n-6]), alpha-linolenic acid (18:3Δ9,12,15 or 18:3[n-3]), and arachidonic acid (20:4Δ5,8,11,14 or 20:4[n-6]) [49]. The FA with cis-double bonds are important in determining the melting points of membrane GPL lipid mixtures, where increases in individual rotational properties and solid-to-liquid phase transitions determine the separation of membrane regions into fluid- and solid-phase domains [49,50]. Lipid lateral phase separations and lipid domain development are important in the formation of membrane rafts and thus, as described above, the establishment of membrane signaling domains [44,45]. Mammalian cells are unable to synthesize or modify certain essential FAs with double bonds at specific positions, and thus some specific unsaturated FA are considered essential dietary constituents [7]. 

The biosynthesis or modification of non-essential FA occurs for the most part in the ER in the cytosol, but some FA can be modified and assembled in the inner mitochondrial membrane [51,52]. This includes some basic GPL construction and modification, such as exchange of phospholipid headgroups and FA sidechains in mitochondria. The complete synthesis of membrane GPL usually follows the sequence: (i) synthesis of a backbone glycerol-3-phosphate molecule, (ii) attachment of FA to this backbone, (iii) dephosphorylation, and (iv) addition of a hydrophilic head group to produce various GPL. Some GPL are synthesized by altering existing GPL molecules, such as phospholipid head group exchange or methylation of ethanolamine groups to form PC [51,52]. Others are modified in their FA side chains, producing an overall membrane GPL composition and saturation necessary for protein remodeling and the formation of lipid rafts and other membrane domains.

The dynamic sorting of membrane lipid and protein components into domains of specific compositions and mobilities was proposed to be primarily based on hydrophobic and some hydrophilic interactions between membrane proteins and lipids [40,45]. Such sorting can avoid hydrophobic mismatches between lipids and proteins, and this can prevent membrane distortions that are unsustainable or eliminate areas of membrane weakness [53]. Thus, the amounts and distributions of GPL and other lipids in biomembranes are important for the structure and physical properties of membranes, but also functional properties related to ion channels, transporters, and receptors. The original Fluid–Mosaic model accounted for basic cell membrane structure, dynamics and asymmetry [42,43], but over time newer models for plasma and intracellular membranes evolved into much more complex and compact structures, but they still retained most of the basic nanometer structure of the Fluid–Mosaic Membrane model [53,54,55,56].

## 3. Mitochondria and Their Membranes

As an essential cellular organelle, mitochondria produce 80–90% of cellular energy and play an indispensable role in redox balance, calcium signaling, energy storage and immune functions, such as innate immunity. They possess a dual membrane structure similar to bacteria, but the critical part of their structure is the mitochondrial inner membrane (MIM) where electron transport and oxidative phosphorylation occur [57,58]. Between the membranes of mitochondria is an intermembrane space, and inside the MIM is the mitochondrial matrix. The matrix contains a mixture of enzymes, mitochondrial ribosomes, tRNAs, mRNAs, and maternally dominant mitochondrial DNA (mtDNA) [57,58,59]. The membranes of mitochondria have discrete lipid compositions that display bilayer asymmetry and lateral heterogeneity. The MIM is the location of the respiratory chain complexes or the electron transport chain (ETC), accessary proteins, and cofactors such as coenzyme Q10 (CoQ10) [58,59]. The ETC essentially pumps protons into the intermembrane space, creating an electrochemical gradient, and the energy stored in the resulting gradient is used to drive oxidative phosphorylation and the synthesis of ATP [22,57,58,60].

Mitochondria have usual membrane concentrations of PE and PC, which are found in all cellular and intracellular membranes. Mitochondria also contain some unique membrane lipids, such as the tetra-acyl phospholipid known as cardiolipin (CL). CL represents approximately 15–20% of the total mitochondrial phospholipid and is found exclusively in the MIM where it is essential in maintaining MIM fluidity and osmotic stability. Along with being functionally indispensable for MIM structure and ETC function, CL is also important in the maintenance of MIM transmembrane potential [22,57,58,59,60,61]. CL molecules are highly sensitive to free-radical oxidation of their FA double bonds, resulting in loss of MIM trans-membrane potential [62,63,64]. The appearance of oxidized CL or its degradation products in blood is also associated with several pathological conditions, including diabetes, heart failure, hyperthyroidism, neurodegeneration, and aging [22]. These conditions are characterized by excess oxidative stress, CL damage, and deficiency, and increases in oxidative products such as docosahexaenoic acid (DHA) [65]. 

In order to function properly, mitochondria must respond to changes in MIM trans-membrane potential. If mitochondria fail to adapt to variations in MIM trans-membrane potential, the electrochemical gradient necessary for ATP synthesis could be lost along with changes in the permeability of several mitochondrial transporters and channels. The loss of the MIM electrochemical gradient creates mitochondrial dysfunction and can eventually result in mitochondrial collapse (mitophagy) [66]. The release of cytochrome c through the BCL-2 proapoptotic channels activates intracellular caspases, leading ultimately to cell death by autophagy [67]. Balancing mitophagy and mitochondrial biogenesis are essential for maintaining cellular homeostasis [68]. A fully-functioning ETC generates an obligatory trans-membrane potential of −150 to −200 mV across the MIM, yielding an equivalent field strength of about 30 million volt/m [69]. Failure to maintain MIM trans-membrane potential results in loss of cellular energy production, increased free-radical ROS leakage, decreased plasma membrane active transport, and suppression of energy-requiring cellular processes. As the MIM is the primary location for production of excess ROS, eventually this can result in cellular stress and affect cellular responses [70].

An increase in intracellular ROS and mitochondrial release of ‘danger signals’, such as damage-associated molecular patterns (DAMPs), pathogen-associated molecular patterns (PAMPs), mitochondrial nucleic acids, and other molecules, are typical of cellular stress responses [70]. Some cellular stresses are also caused by K^+^ efflux, uric-acid crystals and the presence of extracellular ATP, among other stress molecules [71]. The presence of stress agents can provoke the assembly of intracellular multi-protein inflammatory complexes called inflammasomes, which are signaling platforms important in responses against microorganisms and environmental toxins, and this can also result in triggering the secretion of pro-inflammatory cytokines and other cellular responses [70,71,72,73,74]. 

The products of oxidized-unsaturated FA are very important in inducing apoptosis via reaction with mitochondrial permeability transition pores (mPTP). mPTP are voltage-dependent channels that initiate calcium-dependent apoptosis and activate intracellular calcium channels that promote Ca^2+^ release from endoplasmic reticulum and mitochondrial loading of Ca^2+^ [75,76]. Increased mitochondrial ROS production results in oxidation of unsaturated FA and also activates intracellular calcium channels [77,78]. mPTPs are opened during oxidative stress, resulting in Ca^2+^ release and mitochondrial loading that leads to loss of MIM trans-membrane potential and thus reduction in ATP production. The calcium loading of mitochondria further increases ROS production, until mitochondria eventually swell and bleb, degenerate, and undergo mitophagy [79]. Cell death eventually ensues [78]. However, dietary supplementation with unsaturated FA can repair and replace oxidized GPL, modifying mitochondrial unsaturated FA composition and altering mitochondrial calcium homeostasis, thus delaying mPTP damage and Ca^2+^-induced cell death [80].

## 4. Liposomes, Chylomicrons, Lipid Globules, Lipid Micelles, and GPL Transport

MLR GPL taken orally are usually absorbed in the upper small intestines as individual GPL molecules or, if they are degraded, as constituent parts [28,30,31,81,82]. Although some acid degradation of GPL occurs in the stomach, most enzymatic modification takes place in the small intestine. There, FA and other fragments of degraded GPL are transported across the epithelium [81,82,83]. However, when present at high concentrations where GPL are for the most part undegraded, GPL are absorbed as small lipid micelles, globules, and liposomes in an endocytotic process and eventually delivered to the blood circulation [28,29] (see Figure 1 of [1]). This process is also important in the co-transport of other critical molecules that partition into lipid micelles, globules, and liposomes [84]. This evolutionary adaptation is likely present to capture as many GPL molecules as possible, as historically high concentrations of essential GPL were only available at certain times.

**Figure 1:**
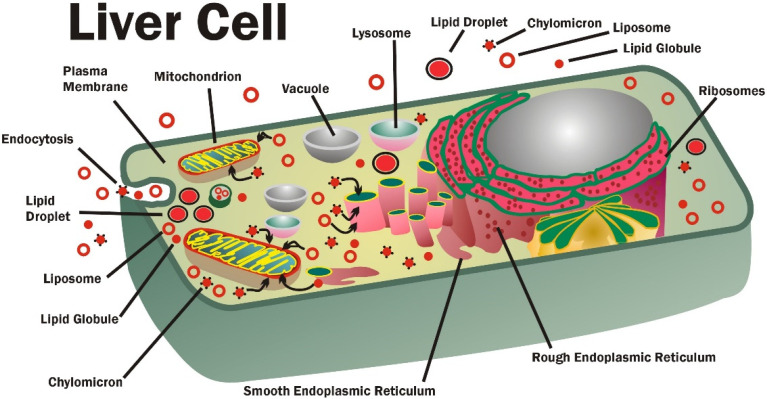
Some of the phospholipid transport systems involved in delivery of MLR phospholipids and other lipids to intracellular membranes and the reverse of this process. A liver cell is shown with internal lipid transport and storage systems, such as micelles, vesicles, globules, chylomicrons, and lipid droplets. These various lipid transport structures can bind to different intracellular membranes and transfer GPL and other lipids, and pick up damaged lipids for delivery outside of cells. Not shown in the figure are lipid transport/transfer by direct adjacent membrane-to-membrane contact and lipid droplet-, globule-, chylomicron- and vesicle-to-membrane contact or temporary fusion with adjacent intracellular membranes. Both the forward and reverse processes appear to be driven by mass action or bulk flow mechanisms. (Modified from Nicolson and Ash [1]).

GPL absorption in the small intestines is an efficient process. After a large meal, over 90% of glycerolphospholipids are absorbed and transported into the blood within six hours [28,83]. In the blood circulation, there is a limit to the amounts of GPL that can be present in carrier molecules, such as lipoproteins, or in the membranes of erythrocytes. However, when present in excess of the amounts that can be carrier-transported, GPL can also be found in blood as lipid globules, liposomes, and other forms (discussed in [1]). Eventually the circulating GPL are delivered to major organs, such as liver (Figure 1), and to cells where they are transferred by direct contact of lipoproteins and erythrocytes with cell membranes or by endocytosis of GPL in micelles, globules, liposomes, chylomicrons, and other forms. They can then transfer to other cells, and the process can be repeated.

Usually, dietary polyunsaturated GPL undergo oxidation and degradation during their storage, ingestion, digestion, and adsorption in the small intestine. Thus, to increase the amounts of intact oral MLR phospholipids that reach their final membrane destinations, they must be protected from oxidation during storage and from acid degradation in the gut, as well as disruption by bile salts and hydrolysis by phospholipases and other enzymes released from the pancreas and gut microflora in the small intestines [1,85]. This has been accomplished by complexing MLR phospholipids with specific fructooligosaccharides, plant inulins which insert between the head groups of glycerolphospholipids and protect them from excess temperatures, acidity, phospholipases, and bile salts [1,86]. The inulins also protect GPL from oxidation and enzymatic degradation [86,87]. When fructooligosaccharides (inulins) are used to protect GPL, MLR supplements can be protected during storage and ingestion, while also being absorbed relatively intact by gastrointestinal brush border cells [1,2]. Although some hydrolysis of GPL will occur during this multi-step process, when MLR lipids are in excess, most of the micellar and globule phospholipids are absorbed by brush border cells as unoxidized, undegraded molecules [1,85].

The uptake of small GPL micelles, globules, or larger droplets by the small intestinal epithelium has been examined using electron microscopy. Morphological studies have shown that undigested dietary lipids and phospholipids present in small intestinal brush border cells are mainly present as small lipid micelles, globules, chylomicrons or larger lipid droplets of 50–1000 Å in diameter [29,88]. When in excess and protected by inulin, the phospholipids are transported by endocytosis or pinocytosis into intestinal cells as largely intact molecules. Even though ultrastructural methods cannot determine the lipid compositions of the ingested lipid globules and droplets, or chylomicrons, these lipid forms are not present in intestinal cells in fasting controls, indicating that they are derived from ingested intestinal GPL [29]. Intestinal epithelial cells can also transport individual GPL molecules and their degradation products, such as FAs, using specific membrane transport systems, but not in the amounts necessary to absorb GPL and other lipids in quantities found during ingestion of MLR supplements. Thus, after routine intestinal cell transport, individual GPL molecules can form micelles, small liposomes, or phospholipid globules, but not in the amounts found after MLR supplementation. It is thought that this routine form of individual molecule transport is less important when GPL are present in excess in the intestinal lumen, where they can associate into small micelles, globules, or larger forms [89] (Figure 1).

Intestinal bush border plasma membranes can directly partition phospholipids into their outer plasma membrane leaflets by membrane contact with GPL. Thus, it was observed that intestinal microvillous plasma membranes became thicker on their outer surfaces during GPL absorption, and this was thought to be due to the direct insertion of GPL molecules into the outer leaflets of brush border microvilli membranes [90]. Once GPL such as PC are enriched in the outer leaflets of the plasma membranes of intestinal cells, they appear to protect this structure from pathogenic processes such as ulcerative colitis and other chronic inflammatory conditions [91]. Individual GPL that are incorporated into the outer plasma membrane leaflet of colonic brush border cells can be transported into these cells by binding to trans-membrane translocases (flipases, flopases, and scramblases) that can transfer the GPL to the opposite membrane surface [92,93]. Once the translocated GPL arrive at the inner plasma membrane surface, they can then be partitioned into protein carriers that transfer the GPL molecules to intracellular membranes or cellular organelles [94]. The build-up of GPL at the inner membrane leaflet may also promote membrane blebs that are then released as new intracellular lipid vesicles and globules [1,29]. Alternatively, GPL can be transferred by partitioning into existing intracellular vesicles, globules, chylomicrons, lipid transfer proteins, intracellular membranes, or other structures and subsequentially transferred to various intracellular sites [1,94,95].

Once GPL and other lipids are moved to various intracellular membrane sites or stored in lipid structures, they can be moved again [95,96,97]. They can also be transferred by membrane–membrane contact. This involves transient membrane fusion or partitioning of lipids from one closely apposed membrane to another membrane or lipid structure, and this type of transfer appears to involve specific membrane transfer domains. For example, ER and mitochondria can transfer membrane lipids by direct membrane contact-transfer through specific ER membrane domains called the mitochondria-associated membrane (MAM) domains [97,98]. Organelles such as mitochondria also have their own specific internal lipid transport systems to move lipids from outer membrane to MIM. Once transfer has occurred, enzymatic alterations can take place, eventually resulting in specific membrane lipid compositions. The reverse of this mitochondrial transport process can exchange damaged membrane GPL with undamaged GPL and removal of the former [99,100,101]. There are also specific lipid transfer proteins in the intermembrane space between inner and outer mitochondrial membranes, and these may also play a role in the maintenance of GPL and FA composition between different mitochondrial compartments [101]. 

The GPL in small vesicles, lipid globules, and chylomicrons can be transferred by direct contact with intracellular membranes or other lipid structures, and eventually this results in partitioning of their contents into various cellular and organelle membrane compartments [1,97,98,102,103,104]. During this process, GPL can undergo enzymatic modifications, such as head group substitution or modification of FA, to reflect the specific membrane compositions at their final destinations. Not much is known about the roles of small GPL micelles, vesicles, and globules inside cells, but additional information has been published on larger lipid structures such as chylomicrons and lipid droplets [105,106]. Intracellular lipid droplets have been defined as structures composed of neutral lipid cores that are surrounded by a layer of PC, PE, PI, and smaller amounts of other GPL and a protein outer surface. The outer surface protein coats of lipid droplets may be used to regulate their size, structure, number, and fate of these lipid storage systems [107]. Lipid droplets are used as the principle lipid storage system in certain cells: adipocytes, hepatocytes, and other cells (Figure 1). They also play important roles in cellular lipogenesis and homeostasis [105,107]. As lipid storage systems, lipid droplets are important in metabolic syndrome (MetSyn), fatty liver diseases, steatohepatitis, atherosclerosis, and other diseases [99,108].

As important cellular lipid storage systems, chylomicrons and lipid droplets are increased to the point of excess during excessive fat absorption and storage, for example, in obesity [106,107]. In contrast to lipid droplets, chylomicrons are mostly GPL (70–75%), with some cholesterol (5–10%), triglycerides (13–25%), FA, and other lipids [109]. Chylomicrons can store some phospholipids in cells, but they are apparently used to transfer lipids to various organelles and different intracellular compartments as well as to adjacent or distant cells [110]. This type of transfer may initially involve small vesicles and globules released from intracellular membranes, such as Golgi membranes, and these can be subsequently formed into chylomicrons. In brush border cells, chylomicrons have been found to be transported to the basolateral surfaces where they are released by a reverse pinocytosis process. Eventually the cell-released chylomicrons find their way to the cells lining the lymph or circulatory systems. At this juncture, endocytosis, transport, and eventually exocytosis can be repeated until these lipid structures are eventually transferred from the lymph and blood to the gastrointestinal system [29,31,110]. In addition to lipid transport in micelles, vesicles, globules, chylomicrons, membranes, and other forms, individual GPL can also be transported by protein lipid carriers or transfer proteins. Preference for particular GPL by protein carriers include differences in GPL head group, saturation, and length of FA acyl chains [94,95,111,112,113]. All of the lipid transport systems appear to function on a mass action or ‘bulk flow’ basis.

As the overall lipid transport process appears to be driven by ‘bulk flow’ or mass action mechanisms to deliver GPL and other lipids to particular membrane sites, the same bulk flow/mass action process can also be used to remove oxidized or damaged lipids from membranes and eventually send them to degradation sites where lipids are degraded or to export sites where they can be exported from cells by exocytosis into the lymph or blood for eventual delivery to the gastrointestinal system for excretion (reviews: [1,2]). 

While in the lymph or blood circulation GPL, steroids, FAs, and other lipids can be transferred to carrier molecules, such as high- and low-density lipoproteins (HDL and LDL), or intercalated into the plasma membranes of blood cells, such as erythrocytes or leukocytes. Circulatory lipoproteins are utilized as an important transport system for lipids in the blood circulation. Once GPL and other lipids are bound to lipoproteins, they are more protected from oxidation and enzymatic digestion during transport. In humans, the amounts of membrane GPL exchanged and preferentially transported by HDL and LDL are more than 20-times the amounts transported by erythrocytes [83]. An added bonus of MLR is that the excess GPL in globules and vesicles can help remove cholesterol from the circulation, in part, by partitioning the GPL into erythrocyte membranes and circulating lipoproteins, and in the process enabling the partitioning and removal of excess cholesterol and oxidized cholesterol [114,115]. Once excess cholesterol is removed from cells in tissues, it can be partitioned into circulating lipoproteins and phospholipid globules and other lipid forms, as well as circulating cells and delivered back to intestinal epithelium for eventual export by the gastrointestinal system [1,3,99,111,114].

As described above, the entire sequence of lipid transport appears to follow a ‘bulk flow’ or ‘mass action’ process along a concentration gradient from the gut to tissues and back again for damaged/oxidized phospholipids [32]. This natural removal process can help slowly reduce oxidized cholesterol and excess cholesterol in membranes, cells, and tissues.

## 5. MLR Formulations

Dietary or oral MLR supplements or intravenous introduction of MLR phospholipids (also called “essential” phospholipids or EPL) have proven to be safe for replacement of damaged membrane lipids, and there is apparently a limit to the amounts of lipids that can be replaced by diet alone. In humans, the maximum total dietary membrane lipids that can be consumed in foods are in the range of 2–6 g per day (reviewed in [6]). For example, legumes are thought to be a good GPL source for dietary MLR supplements [1,2,6]. However, the amounts of, for example, soy beans required to deliver a dose of approximately 1.8 g of membrane GPL would be approximately 15 kg of legumes [116]. Therefore, the consumption of sufficient amounts of plant products to obtain a clinically effective dose of MLR and removal of damaged GPL, sterols, and other lipids would be impractical for most diets [1,2,3]. 

If GPL and other lipids in foods or supplements are unprotected from oxidation, disruption, and degradation, it would be difficult to maintain a clinically appropriate dose level of GPL, which can be several g per day, without contamination of unoxidized GPL with oxidized GPL (Table 1). Protected MLR supplements, on the other hand, can deliver therapeutic doses of GPL, and certain MLR supplements, such as NTFactor Lipids^®^, are fully protected from oxidation, bile disruption, and enzymatic digestion using protective fructooligosaccharides and antioxidants [1,2,3]. MLR supplements containing GPL with n-3 and n-6 polyunsaturated FA and other lipid components have been derived from different sources: plants, fish, and krill, among other sources [1,2,3,6,108,117,118]. These supplements have been tested in laboratory animals and humans for their effectiveness and safety. For example, animals supplemented with n-3 unsaturated FA supplements showed positive changes in mitochondrial membrane phospholipid FA composition, improved mitochondrial function, delayed the opening of Ca^2+^-induced mPTP, and increased mitochondrial reserve [118,119]. Laboratory rats fed for 10 weeks with an unsaturated FA supplement showed beneficial modifications of cardiac mitochondrial CL and improvements in Ca^2+^-induced mitochondrial mPTP function [120]. 

Safe and cost effective MLR oral supplements for humans have utilized various lecithins as a source of GPL [1,2,3,6]. Soy, eggs, or marine krill lecithin preparations have been marketed, but most lack protection from oxidation and disruption by bile or acids. Some oral MLR supplements, such as NTFactor^®^ and NTFactor Lipids^®^, have long shelf-life, are protected by fructooligosaccharides and antioxidants, are safe and contain a full range of GPL, and have been tested for efficacy [1,2,3]. MLR supplements such as NTFactor^®^, which also contains probiotic bacteria, growth media, and other ingredients, and NTFactor Lipids^®^, without the probiotic additives but with fructooligosaccharides and antioxidants, come in several oral forms, and almost all contain 2 g or more of phospholipids per daily dose (reviews: [1,2,3]). 

Other forms of MLR supplements have utilized phospholipid-specific preparations. For example, PS supplements have been used for specific purposes, such as to treat memory loss in aged subjects or in Alzheimer’s disease (AD) patients. Supplementation with 300 mg per day of bovine PS for 6 months resulted in cognitive improvements in AD patients compared to controls [121]. However, another study in elderly subjects with age-associated memory impairment that received 300–600 mg PS daily for 12 weeks did not show memory improvements [122]. Although supplementing with a single class of glycerolphospholipid alone, such as PS, has been shown to have health benefits, the use of more complex mixtures of membrane GPL may be more beneficial, possibly as some of the PS does not have to be enzymatically converted to other GPL [1,2,3,123,124]. 

In addition to oral supplementation, MLR phospholipids have been introduced intravenously in hospital or out-patient settings [6]. Acute cases of liver toxicity, kidney failure, hepatitis, dialysis, and other conditions have been treated with intravenous EPL [6]. Administration of EPL phospholipids IV have been used to deliver high concentrations of GPL without the need for fructooligosaccharides to inhibit intestinal disruption. Any intestinal adverse events can be minimized by IV administration, but it is expensive compared to oral supplementation, and administration must be professionally supervised. GPL IV preparations, such as Essentiale^®^, contain approximately 1 g of mainly PC (>75%) with some minor amounts of other GPL and other ingredients in a single IV treatment [6]. 

## 6. Safety of MLR

MLR oral supplements are extraordinarily safe [1,2,3,6]. Preclinical and clinical safety studies have not shown any acute or chronic MLR supplement toxicity, even at very high daily doses levels. There has been no evidence of MLR perinatal and postnatal toxicity; nor were there any mutagenic or carcinogenic effects of administering GPL. None of the preclinical studies conducted in laboratory animals (mice, rats, and rabbits) demonstrated any acute or chronic toxic effects of oral, subcutaneous, or intravenous GPL administration (review: [1]). In vivo studies in laboratory animals using potentially toxic or lethal doses could not establish any toxic or lethal dose levels for MLR supplements. Doses up to 45 g of MLR GPL have been given orally without evidence of any adverse effects [125]. 

Using mutagenicity and carcinogenicity animal models the effects of escalating doses of MLR phospholipids were completely negative. For example, animals fed daily oral doses up to 3.75 g/kg body weight produced no measurable toxic, mutagenic, or carcinogenic events (review: [1]). In addition, GPL dose toxicity could not be established in young, adult, pregnant, or fetal laboratory animals. Additionally, evidence of any GPL toxicity was not found in pregnant rats or rabbits or in their newborns at doses up to and above 1 g/kg (review: [1]). In addition, the co-administration of MLR GPL and carcinogens in laboratory animals revealed no evidence that MLR can increase carcinogenicity. In fact, MLR with GPL actually inhibited the formation of tumors in animals [126]. 

The effects of MLR on animal health has also been examined in life-long studies (review: [1]). GPL have been given in chow or water to laboratory animals at daily doses from 0.01–5 g/kg body weight, and there were no differences between test and control animals in central or peripheral nervous systems, renal function, heart, and vascular function, or other indicators of toxicity (reviewed in [1]). Lifetime administration of GPL to laboratory rodents has demonstrated only beneficial effects. For example, feeding rats NTFactor^®^ in their chow over their lives reduced age-related hearing loss and deceased mtDNA deletions associated with aging [127]. In addition, aged rats were fed NTFactor^®^ or placebo for 6 months and their auditory brainstem responses, MIM potentials, and mitochondrial DNA deletions were examined every 60 days. Auditory brainstem responses were estimated by measuring hearing thresholds, and MIM membrane potentials were estimated with redox dyes using blood leukocytes. DNA deletions in the aged rodents were determined by extracting mtDNA from various brain regions and amplification of mtDNA sequences. Deletions of known mtDNA sequences lost during aging were verified [127]. There were significant differences between the groups of animals receiving MLR GPL and placebo groups in terms of auditory brainstem responses, MIM potential and the presence of mtDNA deletions. With administration of NTFactor^®^ in the chow of the rats, there was significant preservations of hearing threshold at all frequencies tested in the experimental animal group. NTFactor^®^ also prevented the usual age-related decline in MIM trans-membrane potential and reduced the incidence of common mtDNA deletions in the aging rats [127].

High MLR doses of GPL have been taken orally by humans with no evidence of any toxicity or adverse events (review: [1]). In cases of hepatic encephalopathy due to decompensated liver cirrhosis, patients receiving EPL showed significant improvements in their liver disease status and had prolonged survival compared to a control group that did not receive EPL [126]. The patients in this study also showed no evidence of any toxic effects from the GPL. The use of high doses of MLR GPL in long-term studies in humans has shown that subjects actually showed improvements in cardiovascular blood markers. In one study, 35 elderly subjects received at least 4 g per day oral NTFactor^®^ over 6 months. During this period participants showed no evidence of any adverse events, and their blood cardiovascular risk markers, such as homocysteine, improved. In some patients, homocysteine levels decreased from high-risk levels to normal ranges [128]. In a different study, 58 middle-aged patients with fatiguing illnesses received doses of 4 g per day oral NTFactor^®^ for 2 months without any adverse incidents [123]. Some of these patients continued using the MLR supplement for years without reporting any adverse events. The long-term use of MLR GPL in clinical studies on cardiovascular diseases has been reviewed, and there is no evidence of MLR GPL toxicity [129]. Safety studies, along with the lack of observed toxicity in clinical trials likely supported the decision of the U.S. Federal Drug Administration (FDA) classify oral MLR GPL as GRAS or ‘Generally Recognized as Safe’ [130]. To date, there have been well over a million doses of MLR supplements taken with no reported adverse effects.

## 7. MLR in Aging

The aging process is thought to be linked to the progressive accumulation of physiological changes, such as declining cognitive and immune function, reductions in cellular activities due to accumulations of cellular damage, and enhanced likelihood of contracting chronic and lethal diseases. The foundations of normal aging appear to be complex, with no single mechanism explaining every aspect of this complicated phenomenon. There are multiple effects of aging that can be seen at the cellular level. Some age-related changes have been related to genomic instability, telomere shortening, mitochondrial dysfunction, cellular senescence, epigenetic alterations, dysregulated nutrient sensing, stem cell exhaustion, altered intercellular communication, changes in calcium stores, and inflammation, among other changes [131,132,133,134]. One characteristic of human aging is the change in a type of inflammation—chronic, progressive inflammation—that is related to mitochondrial responses [135]. These types of inflammation responses are often linked to age-related diseases and disorders [133,136]. 

Mitochondria are especially important in aging, and their decline in function and numbers with age involves a number of possible mechanisms, including: (i) increased mitochondrial uncoupling and production of ROS; (ii) insufficient antioxidative enzymes to counter the increased production of ROS; (iii) increased disorganization of mitochondrial structure, especially mitochondrial membranes; (iv) changes in intracellular Ca^2+^ stores; (v) decline in mitochondrial oxidative phosphorylation and production of ATP; (vi) accumulation of mtDNA deletions and mutations; and (vii) increased damage to mitochondrial proteins and lipids [1,131,132,133,134,135,136,137,138,139,140,141,142,143]. These events are not mutually exclusive and likely contribute to the overall decline in mitochondrial number and function with age.

An aspect of aging relating to cellular organelles, such as mitochondria, is the accumulation of endogenous debris, such as age-related accumulation of membrane and mitochondrial debris. This accumulation may reduce the ability of mitochondria to remove the debris by mitochondrial autophagy and mitophagy [132,136]. The accumulation of endogenous debris can lead to protein aggregation and accrual of damaged proteins that eventually results in reductions in mitochondrial function. Some of the observations on aging and mitochondrial function, such as age-related reductions in oxidative phosphorylation, reduced MIM trans-membrane potential and increased permeabilization of the outer mitochondrial membrane, among other changes, are consistent this hypothesis [135,140]. In addition, the increases in ROS/RNS production and resulting oxidative stress found with aging cause further oxidative damage, causing reductions in membrane fluidity and damage to mitochondrial CL due to lipid peroxidation [141,142,143]. The age-related functional decline seen during aging, along with changes in many other markers of aging could also be related, to a large degree, to losses in mitochondrial function and number [141]. An obvious example is fatigue. Aged individuals universally complain about this problem [144]. The role of age-related membrane changes to fatigue will be considered in more detail in Section 8. 

MLR can be used to modify the usual decline in mitochondrial function seen during aging. In one clinical study on aging, daily use of MLR with NTFactor^®^ resulted in improved MIM trans-membrane potential, which has been linked to mitochondrial function, reduced fatigue, and increased cognition measured using a validated survey instrument [145]. The use of oral MLR and the improvements in MIM trans-membrane potential suggest that mitochondrial membranes were functionally improved in aged individuals by oral MLR supplementation. Interestingly, the MIM trans-membrane potential in the aged group was improved over time and actually reached the levels found in healthy 30-year-olds [145]. MLR supplementation has also been used to improve cognition in aged individuals. Daily oral administration of 300 mg of PS in aged patients was found to deliver significant improvements in cognition compared to controls [121].

Multiple clinical studies have linked the process of aging to mitochondrial decline. For example, studies have shown that aging is inversely related to the cellular content and number of mitochondria and maintenance of their function [146]. The loss in the number of mitochondria in each cell correlated with age along with loss of mitochondrial CL content and changes in membrane fluidity due to lipid peroxidation. This latter process affects MIM trans-membrane potential, which requires an appropriate redox balance to be fully functional, and this is a significant challenge during aging [147]. These changes result in total cellular reductions in ETC activity and ATP production [134,136,141,142]. Nonetheless, such changes can be ameliorated by MLR interventions. Indeed, patient improvements in subsets of mitochondrial FA oxidation disorders have been seen during MLR interventions [147]. The regulation of the biosynthesis and degradation of GPL in mitochondria, and their transport into and out of the outer and inner mitochondrial membranes play important roles in maintaining cellular health and delaying age-related declines in mitochondrial function. As the mitochondrial production of ATP is directly linked to the regulation of the synthesis, degradation, and transport of GPL and of maintenance of MIM trans-membrane potential [148], the provision of fully-functional GPL substrates by the use of oral MLR supplements or nutritional means represents a safe option for dealing with age-related mitochondrial decline [1,2,3]. 

The use of MLR to inhibit CL peroxidation and its cellular relocation, as well as MLR to provide precursor molecules for CL synthesis, can also reduce the risk for age-related damage to mitochondria. In addition, CL is also involved in the import and assembly of mitochondrial proteins [149], and the majority of such mitochondrial proteins are nuclear-encoded and require protein translocases to be transported to their mitochondrial membrane targets. This transport system relies on ATP, and mitochondria provide 80–90% of the cellular ATP necessary for cellular function, including membrane function and reorganization [134,150,151]. 

Another age-related process affecting cells and originating within mitochondria is programmed cell death. This involves the specific, organized destruction of mitochondria known as mitophagy, which in turn is highly sensitive to redox potential and the concentrations of GPL in mitochondrial membranes [151,152,153]. Some age-related changes are, in turn, related to the accumulation of dysfunctional mitochondria within cells, resulting from a combination of impaired clearance by autophagy and inadequate mitochondrial replenishment [153,154]. The reduced regenerative capacity or turnover of healthy cells and mitochondria in this state can be the result of mtDNA damage and mitochondrial dysfunction, further adding to the vicious cycle of inflammation and age-related disease onset [155]. For example, increased production of ROS/RNS associated with aging can stimulate NLRP3 inflammasome formation by reacting with mtDNA, and this can promote chronic inflammation [156,157,158,159,160]. Although ROS/RNS are also important in regulating and maintaining normal homeostatic processes in living organisms, their excess during aging can lead to sustained inflammasome production of IL-1β and IL-18 [160,161]. MLR with dietary FA can affect the over-expression of inflammasomes during aging. For example, n-3 unsaturated FA, such as DHA and EPA, exhibit anti-inflammatory properties by inhibiting the production of inflammasomes [162,163]. Multiple mechanisms may be involved, including the manipulation of cell membrane lipids as well as inhibiting primary and secondary factors, such as modifying Ca^2+^ stores, the expression of NFκB, and modifying cholesterol metabolism [164,165]. Using MLR dietary lipids to inhibit chronic inflammation may be useful in reducing age-related chronic inflammation and maintaining a functional balance between inflammatory and anti-inflammatory responses.

Mitochondria can be functionally improved by the several cellular mechanisms, including mitochondrial fission and fusion, and their numbers can also be increased by mitochondrial biogenesis [166,167]. Mitochondrial fission is normally associated with DNA replication and is essential for mitochondrial duplication and biogenesis. Mitochondrial fission is also essential for mitophagy, the recycling of mitochondria that have become dysfunctional or damaged [168]. Mitochondrial fusion can repair damaged mitochondria and maintain their function [166,167,168]. It can occur rather quickly, and it is mediated by a three-stage process, involving: (i) end-to-end alignment of mitochondria; (ii) fusion of the outer membranes of mitochondria; and (iii) fusion of the inner membranes, forming a larger intact mitochondrion [169,170,171]. Mitochondrial fusion can rescue impaired mitochondria by reorganizing their contents and unifying the mitochondrial compartment [172,173]. MLR can be used to help support mitochondrial fission and fusion and to increase ATP production, decrease membrane permeability, increase MIM trans-membrane potential, reduce inflammasome production and eventually improve organ function [124,126,145,164,165,166,167,168].

How cellular function can be enhanced by MLR supplementation to improve age-related changes has been visited in a randomized, double-blind, placebo-controlled study in middle-aged, pre-, and post-menopausal women who complained of some fatigue but were relatively healthy [174]. The participants (32 patients in each arm) were given MLR with NTFactor (0.8 or 1.2 g per day or placebo) for 4 or 8 weeks, and fatigue, vigor, mood, and menopause symptoms were monitored. The women receiving 1.2 g per day GPL showed greater reductions in fatigue (compared to placebo) using the Chalder fatigue scale but this did not reach significance. There were significant improvements in patient vigor in the GPL group. Sleep, confusion, anger, and menopausal symptoms were evaluated using Quality of Life instruments, and were found to improve, but again this did not reach significance. Cardiovascular parameters improved, and reductions in diastolic blood pressure and improvements in cardio-ankle vascular index were significant [174].

MLR use, either as a sole supplement or as part of a nutrient and lifestyle intervention, including fasting, exercise, and increased intake of plant-based foods, can improve membrane functionality and mitochondrial function, and make meaningful improvements in age-related disorders [1,2,3,121,175,176,177,178,179]. Some elements of functional decline associated with aging appear to be more susceptible to MLR supplementation than others. For example, there are particular morbidities such as elements of coronary heart age-associated diseases, stroke, neurodegeneration and some metabolic disorders, that are at least partially to MLR used either as a stand-alone supplement to improve outcome and reduce age-related morbidity or as part of comprehensive program of dietary and lifestyle changes. 

## 8. MLR in Fatiguing Illnesses

Fatigue is a widely found indicator or symptom in various diseases, conditions, and during normal aging, but it nonetheless remains poorly understood. It is a complex, multi-dimensional sensation that is perceived as loss of overall energy and feeling of exhaustion and inability to perform tasks without excessive exertion [144,180,181,182]. Mild fatigue can be attributed to several mental and physiological disorders, such as depression, but moderate to severe fatigue is generally related to loss of mitochondrial function and reduced production of cellular ATP [124,175,182,183]. 

Mitochondrial stress, and especially damage to mitochondrial membranes by ROS/RNS, occurs naturally during aging and in essentially all chronic and acute medical conditions [1,124,182,183,184]. Patients with severe fatigue, such as in chronic fatigue syndrome or myalgic encephalomyelitis, evidence of free-radical oxidative damage to the DNA and lipids, especially membrane and mitochondrial lipids, is commonly found [184,185]. Patients with severe fatigue also test positive for oxidized blood markers^,^ such as oxidized membrane lipids, that are indicators of excess oxidative stress [186,187]. Patients with chronic fatigue syndrome also have sustained elevated levels of peroxynitrite due to excess release of nitric oxide. An overabundance of peroxynitrite also results in excessive lipid peroxidation and loss of mitochondrial function. Peroxynitrite is a potent stimulator of increases in cytokine levels that can have a positive feedback effect and increase nitric oxide production even further [188].

As the most commonly found symptom in chronic medical conditions, fatigue is also one of the most familiar symptoms seen in cancer patients [12,135,136]. In cancer patients, fatigue can occur at the onset of disease, and it is certainly present in the most progressive forms of metastatic disease [189,190]. The most universal symptoms found in cancer patients are fatigue, pain, and nausea, and these are often found as disabling symptoms [189,190]. Similar to fatigue found in other diagnoses, cancer-associated fatigue is also not well understood. It is thought to be a combination of the effects of the cancer plus the effects of cancer treatments. Most cancer therapies are known to cause fatigue [189,191].

Until recently cancer-associated fatigue was largely considered to be an untreatable condition and was thought to be unavoidable [189,192]. As cancer has often been found to be associated with symptoms of depression and anxiety [193], cancer-associated fatigue was believed to be a psychological problem that was only secondarily associated with cancer [191,192]. Indeed, malaise, fatigue, and even loss of vitality were considered associated characteristics with depression, and fatigue and its manifestations are now considered primary elements in the diagnosis of depression [193,194]. As stated above, fatigue and depression are often diagnosed together in cancer patients, especially in patients with severe disease; thus, they can be considered cancer co-morbidities or as part of a symptom cluster in cancer patients [194]. 

Therapy-associated fatigue is often seen during the treatment of cancer, and such treatments often utilize techniques that result in the generation of free radical oxidants [189,191,195]. Fatigue can be so severe during cancer therapy that it is often the most common reason why patients discontinue their therapy [191]. Up to 96% of patients receiving chemotherapy and up to 93% receiving radiotherapy recount moderate to severe therapy-associated fatigue, and such fatigue can continue for months or even years after the completion of therapy [196].

MLR can lessen cancer-associated fatigue and reduce the fatigue caused by cancer therapy [189,195,197,198,199,200,201,202,203,204,205,206,207,208,209,210]. MLR supplements, such as NTFactor^®^, reduced cancer-associated fatigue approximately 30–40% [189,197,198,199]. In addition, MLR supplements have also been used to reduce the adverse effects of cancer therapy, such as fatigue, nausea, vomiting, malaise, diarrhea, headaches, insomnia, constipation, and other adverse events [189,198,199]. Using a combination MLR supplement mixture containing NTFactor^®^ Colodny et al. [199] were able to reduce several common adverse events during and after cancer chemotherapy of colon, rectal, and pancreatic cancers. MLR supplements, such as Propax^TM^ with NTFactor^®^, have also been used to reduce the adverse effects of treatment using multiple chemotherapy agents. MLR supplementation resulted in significantly fewer episodes of fatigue, nausea, vomiting, diarrhea, constipation, insomnia, and other effects. Cancer patients on combination chemotherapy that used the MLR supplement NTFactor^®^ also experienced an overall improvement in quality of life indicators. In a double-blind, placebo-controlled, cross-over study on patients with advanced cancers undergoing combination chemotherapy a MLR supplement containing NTFactor^®^ was used. During anti-cancer therapy patients had fewer and less severe adverse effects due to the combination chemotherapy. For example, there were improvements in the incidence of fatigue, nausea, diarrhea, impaired taste, constipation, insomnia, and other symptoms compared to controls [199].

Lipids are important in cell proliferation, and thus their use in cancer therapy and support of cancer patients has been considered [189,198,199]. There are over 100 clinical cancer trials that have used the polyunsaturated fatty acids EPA and DHA, and over 60 trials have used other fatty acids and phospholipids (Clinicaltrial.gov website, accessed on 1 November 2021). For example, the efficacy and safety of 2-hydroxyoleic (LAM561) and 2-hydrocylinoleic (ABTL0812) acids have been demonstrated in clinical trials (Clinicaltrials.gov identifiers #NCT01792310 and #NCT03366480, respectively). They exert their MLR effects after their incorporation in cell membranes, both in the original forms or via their metabolites, and they induce relevant changes in the interactions of membrane proteins that regulate cell signaling [200] in ways that reduce cancer cell proliferation and induce autophagy [201,202].

The most common clinical use of MLR with GPL is to treat fatigue not associated with depression or cancer. For example, MLR has been used in several studies in patients with fatiguing illnesses to reduce moderate to severe chronic fatigue. Patients complaining of fatigue lasting more than 6 months are considered patients with chronic fatigue [123,124,145,175,189]. The effects of NTFactor^®^ on chronic fatigue in moderately fatigued subjects were documented to determine if mitochondrial function improved simultaneous with reductions in fatigue in patients taking oral NTFactor^®^ [145]. In this cross-over clinical study, there was good correspondence between the reductions in fatigue and gains in mitochondrial function as determined by increases in MIM trans-membrane potential in leukocytes using a redox dye and fluorescent-activated cell sorting. As discussed above, mitochondrial function is directly related to MIM trans-membrane potential. After 8 weeks of MLR with NTFactor^®^, mitochondrial function had improved significantly, and after 12 weeks of NTFactor^®^ supplementation, mitochondrial function was found to be similar to the levels found in young healthy adults [145]. Specifically, there was a significant increase (+26.8%) in mitochondrial function, measured by increases in MIM trans-membrane potential. After 12 weeks of supplement use, subjects were switched from MLR to placebo without their knowledge for an additional 12 weeks, and their fatigue and mitochondrial function were again measured. After the 12-week placebo period, fatigue and mitochondrial function were intermediate between the initial starting values and those found after 8 or 12 weeks on the MLR supplement, suggesting that continued MLR supplementation is probably required for further improvements in mitochondrial function and maintenance of lower fatigue scores [145]. Similar results on the effects of NTFactor^®^ on fatigue were found in patients with chronic fatigue syndrome (CFS/ME) and/or fibromyalgia syndrome, Gulf War Illness, chronic Lyme disease, mycoplasmal infections, or various other conditions [123,145,175,203,204,205].

MLR combination supplements have proved advantageous and safe for treatment of fatiguing illnesses [1,2,3,123,145,175]. MLR supplements containing NTFactor^®^ have also been used in combination with other ingredients to treat long-term chronic illness patients with moderate to severe fatigue [3,133,203,204,205]. In one of these studies, patients had been ill with intractable fatigue for an average of over 17 years and had been seen by an average of more than 15 physicians. In addition, they had taken an average of over 35 supplements and drugs with no improvements in their fatigue scores [123]. Within a few weeks on the combination MLR supplement ATP Fuel^®^, they responded and showed significant reductions (30.7%) in fatigue. Regression analysis of the data indicated that the reductions in fatigue were consistent, occurred with a high degree of confidence (R^2^ = 0.960 for overall fatigue) and were gradual. Thus, the ATP Fuel^®^ proved to be a safe and effective in significantly reducing fatigue in patients with intractable chronic fatigue [123]. A recent follow-on, open label study using fewer subjects and a second-generation ATP Fuel^®^ supplement (ATP360^®^) found similar results [206]. Chronic fatigue was reduced within one week and the results were statistically significant throughout the 8-week study. They also monitored blood pressure and found that the ATP360 supplement reduced significantly diastolic blood pressure and pain (discussed below). Wellness scores were collected during the study, and participants on ATP360^®^ were found to have significant improvements in sleep and vitality and mental function scores by the end of 8 weeks. Cytokine TNFα in serum was reduced significantly by week 4. They also examined mitochondrial resistance to inflammatory stress ex vivo in blood leukocytes from chronic fatigue patients and controls treated with the endotoxin lipopolysaccharide. After 4 weeks consumption of the combination supplement, leukocyte subpopulations responded to the ex vivo inflammation stress by producing a similar increase in mitochondrial mass per cell as leukocytes from healthy controls, and monocyte trans-membrane MIM potential increased by 51% in cells from chronic fatigue patients, but this was not statistically significant [206]. 

Another type of oral MLR combination supplement containing NTFactor Lipids^®^ and low-dose, controlled-release caffeine was used to reduce fatigue, pain (to be discussed below), gastrointestinal symptoms and improve Quality of Life (QOL) indicators in fibromyalgia patients [207]. Previously, it was noted that low-dose caffeine, equivalent to 1–2.5 cups of coffee, had a modest effect in reducing some chronic pain and some fatigue, but among non-opioid users these effects were not significant [208]. By combining NTFactor Lipids^®^ plus low-dose, control-release caffeine (equivalent to one cup of coffee maintained over 8 h) in a chewable wafer a preliminary open label study was initiated to study its effects in group of fibromyalgia patients during an 8-day open-label trial. By the end of the study patients showed significant reductions in fatigue, pain, and gastrointestinal symptoms and improved abilities to complete tasks and participate in activities (Quality of Life indicators) [207]. 

Mitochondria-supporting combination supplements have been used to treat mitochondrial cytopathies and to support mitochondrial function [123,124,205,206]. The use of combination supplements containing antioxidants, alpha-lipoic acid, vitamins, CoQ10, lycopene, creatine, riboflavin, and other ingredients have been proposed [209]. Gonzales et al. [210] have discussed in detail the use of various ingredients, including MLR, in supporting mitochondrial function and improving health outcomes.

## 9. MLR in Pain Control

Chronic pain is a complex symptom involving nerve membrane channelopathies (damage to membrane channels in nerve cells that initiate the transmission of pain signals), autoimmune responses, microvascular damage, and other factors [211,212]. There are several types of pain, but the chronic pain under consideration here is nociceptive pain, which is pain originated by depolarization of nociceptors in sensory nerve fibers in response to noxious stimuli [213]. In terminal nociceptors noxious stimuli can elicit membrane currents that, if they surpass a certain threshold, send signals from the nerve fibers to the spinal cord. The conducting fibers from the periphery to the Central Nervous System involved in pain perception are usually of the A or C type [213]. MLR supplements such as NTFactor Lipids^®^ have been used to reduce widespread and chronic pain in patients [214,215]. The MLR lipids are not thought to be acting by repairing mitochondrial membranes; they are thought to act by modulating and restoring the functions of cell membrane ion channels (for example, TRPV, P2X, or ASIC channels), maintaining intracellular Ca^2+^ concentrations or other effects [213,216]. Indeed, nerve cell membrane ion channels related to pain pathways can be dramatically modulated in their function by particular MLR GPL [216,217]. In addition, other properties, such as stimulation of free radical oxidative damage, loss of mitochondrial function or the presence of proapoptotic enzymes such as caspases, could also be modulated in nerve cells by MLR GPL [218]. 

MLR supplements, such as NTFactor Lipids^®^, have been used to reduce pain, such as widespread pain, gastrointestinal pain, and other symptoms in chronic illness patients. For example, in fibromyalgia patients 4.8 g per day of oral NTFactor Lipids^®^ for 8 days significantly reduced pain (*p* < 0.001), fatigue (*p* < 0.001), gastrointestinal symptoms (*p* < 0.001) and improved Quality of Life assessments (*p* < 0.001) in an open label clinical trial [207]. The MLR supplement NTFactor Lipids^®^ has been shown in case studies to reduce widespread pain in fibromyalgia, gunshot wounds, and other chronic conditions [214,215]. This new area of MLR use will continue to grow in the future, but there is one important limitation. The pain responses to MLR appear to be limited to higher daily doses of MLR supplements. Patients responded better and their responses were more durable at higher daily doses (≥6 g per day) of the MLR supplement. For example, oral NTFactor Lipids^®^ was found to be most effective at reducing widespread pain, specific musculoskeletal pain, abdominal pain, or other pain if used at or above 6 g per day. In these case studies, the MLR supplement also showed benefits in sleep, digestion, hypertension, headaches, and other signs and symptoms [214,215]. This dose requirement may reflect the concentrations of GPL necessary to modulate membrane channels that mediate pain transmission or other mechanisms. As found in previous MLR studies, when the higher daily oral doses of NTFactor Lipids^®^ were lowered or stopped, widespread and other pain as well as other signs and symptoms returned to levels found before MLR supplementation, consistent with the bulk or mass action delivery of MLR to peripheral and central nerve cells and other cells involved in pain transmission. 

## 10. MLR in Degenerative Diseases

As mentioned above, the proper maintenance of mitochondrial health is essential in order to prevent the formation of degenerative diseases [210,219,220,221,222,223]. Non-infectious, non-communicable degenerative diseases (NCD) are age-related diseases whose incidence increase with age or certain behaviors. The most common NCD are: cardiovascular diseases (CVD) (hypertension, cardiopathies, coronary heart disease, myocardial infarction, and stroke), neoplastic diseases (various cancers), neurodegenerative diseases (Alzheimer’s disease, Parkinson’s disease, and multiple sclerosis, among others), autoimmune disease, rheumatic diseases, and chronic respiratory diseases. Mitochondrial changes, such as mitochondrial fission and fusion to improve function, play critical roles in ensuring and maintaining mitochondrial health. However, if high concentrations of fats or other nutrients are consumed in excess, molecular damage may occur that can spread through mitochondria and affect their collective performance [224]. Mitochondria can optimally produce ATP when they are undamaged or only marginally damaged, or alternatively if the damage is repaired, for example with MLR. When mitochondria are damaged by free radical oxidants, their dynamics can be unfavorable, and fission and fusion often fail to recover function. This suggests that nutritional support and lifestyle changes that favor optimal mitochondrial dynamics, for example, with appropriate MLR, antioxidants, and other nutrients along with caloric restriction, can result in enhancement of mitochondrial function, increased cellular energy, and improved health [225,226]. Defects in mitochondrial membranes, systemic inflammation, and oxidative stress are thought to be the most likely causes of most NCD. Therefore, nutritional interventions, repair of mitochondrial damage, and improvements in lifestyle have the potential to improve health [226,227,228]. 

Nutritional, pharmacological, and lifestyle approaches can potentially slow down age-related functional decline and damage that predisposes an individual to NCD [220,225,226,227,228]. Such approaches include the use of insulin sensitizers [229], exercise to promote mitochondrial fusion [230], and targeted antioxidant treatments to reduce ROS/RNS damage to membranes [231]. However, the effectiveness of many nutritional approaches, such as the general use of antioxidants, has been questioned [231]. Most antioxidants are not selective for mitochondria, and they fail to complex with lipophilic transporters and do not reach the MIM [232]. Other possible therapeutic approaches, such as caloric restriction or reduction in caloric intake without malnutrition or intermittent fasting continue to be examined [233,234,235]. Use of combination supplements with protected GPL and antioxidants has been shown to improve mitochondrial bioenergetics [1,2,3,12,124,145]. This has the added benefit of potentially reducing inflammation [206]. Unfortunately, high caloric western diets, with excessive consumption of sugar and saturated fats, along with environmental insults, can result in mitochondrial dysfunction and higher susceptibilities to NCD, premature aging, and carcinogenesis [233,234,235,236,237]. Therefore, lifestyle and behavioral changes combined with nutrition and MLR can provide essential mitochondrial support and may prevent or forestall the development of NCD [238,239]. 

One of the pathways involved in regulating mitochondrial function involves the enzyme ‘mammalian target of rapamycin’ (mTOR), a serine/threonine kinase that regulates cell growth responses to nutrients [240]. There are two important mTOR complexes, and dysregulation of their activities is associated with aging and various NCD, including diabetes, cancers, and neurodegenerative diseases [241]. Signaling by one of these complexes (TORC1) regulates mitochondrial biogenesis, oxidative stress, and turnover in mammals and lower organisms. Defects in the clearance of damaged mitochondria by TORC1-regulated autophagy contributes to accumulation of cellular ROS/RNS [242]. The TORC1 signaling pathway is involved in the sensing the abundance of various nutrients and regulating the activity of critical processes, such as autophagy. Caloric restriction without malnutrition and periodic short-term nutrient excess with use of specific inhibitors of mTOR can affect mitochondrial function [243,244]. This suggests that mitochondria are highly responsive nutrient sensors and effectors, and this could potentially be used to suppress the generation of NCD.

The use of MLR along with lifestyle changes may present an opportunity to enhance mitochondrial fusion, while reducing inflammation and oxidative stress, and this could ultimately assist in the management, prevention, and treatment of NCD. As an example of this approach, a preliminary clinical trial in which MLR supplementation was used to modify metabolism through weight reduction and appetite restraint, was initiated [245]. Thirty middle-aged adults (mean age, 56.8; 24 females and 6 males) used an oral MLR supplement (HealthyCurb^TM^, containing NTFactor^®^ and alpha-amylase inhibitor) 30 min before each meal. Participants were told to eat and exercise normally. In the study, weight, waist, and hip measurements were taken weekly, and blood samples were taken prior to and at the end of the study for lipid and chemical analyses. Appetite and sweet cravings were assessed weekly by standard methods, and fatigue was determined using a fatigue instrument. A majority (63%) of the participants lost an average of 2.77 ± 0.12 kg, along with significant average reductions of 6.4 ± 0.13 cm and 3.8 ± 0.10 cm inches from waist and hip circumferences, respectively. The entire group lost an average of 1.65 ± 0.11 kg (*p* < 0.001) with significant average reductions of 4.04 ± 0.06 cm and 2.87 ± 0.05 cm in waist and hip circumferences, respectively. Weight loss and body measurement decreases were gradual, consistent, and significant, along with reductions in body mass index and basal metabolic rate. Hunger was significantly reduced (44.5%), and there was decreased cravings for sweets and fats. There was a 23.9% reduction in fatigue during the study. There was also a 26.8% improvement in cognition as well as improvements in the ability to think clearly, concentrate, and remember details. At the end of the study blood lipid profiles showed improvements, with better cardiovascular lipid profiles (lipoprotein ratios). During and after the study no adverse events were reported [245]. The effects of MLR on weight loss in the clinical studies could be due to the replacement of damaged GPL with undamaged GPL and support of mitochondrial function. In animal weight-loss studies, rats were fed a diet high in stearic, elaidic, oleic, linoleic, and 2-hydroxyoleic acids for 7 days. On this diet, rats showed less food intake and there was weight loss, mainly due to reductions in adipose fat [246]. Subsequently, they found that only the chow containing excess oleic acid or 2-hydroxyoleic acid induced body weight loss [246]. The clinical trial and animal study indicated that MLR supplements can reduce the urge to eat, and this may be related to their positive, systemic effects on mitochondrial function.

MLR with GPL have been used to modify NCD-related functional decline in patients, such as supplementation with oral PS to improve memory loss and cognition. For example, in one 12-week study 30 male and female subjects (age 50–90 years) with memory impairments not related to neurological disease, stroke, intracranial hemorrhage, brain lesions, diabetes, infections, or inflammatory processes were used to ascertain if 300 mg PS per day modified the outcomes of 6 tests of memory and cognition [247]. At the end of the study participants exhibited significant improvements in memory recognition, recall, total learning, executive functions, mental flexibility, and visual spatial learning. Participants also showed reductions in mean systolic and diastolic blood pressure compared to baseline pressures, and there were no adverse events reported [247]. Similarly, a double-blind, randomized clinical trial on 78 subjects (50–69 years) was initiated to determine if 100–300 mg oral PS per day versus placebo affected memory. In this study, PS supplementation significantly improved behavioral memory functions, especially short-term memory and cognitive function (measured by delayed word recall). The study was completed without any adverse events or changes in vital signs or laboratory tests [248]. The clinical results using MLR supplements universally did not result in any adverse events.

The studies discussed above support the relevance of membrane lipids in the pathophysiology of neurodegeneration. In this context, recent studies demonstrate that Alzheimer’s disease is instigated, in part, by alterations in membrane lipids that alter the functions of important membrane proteins [249]. Thus, membrane GPL and other membrane lipids regulate the nanodomain localization and functional activity of many if not most membrane proteins, and changes in lipid species present in the plasma membrane appear to be associated with mild or severe deteriorations associated with pathophysiological cognitive decline [250]. In this context, treatment with 2-hydroxydocosahexaenoic acid (DHAH) induced marked increases in the levels of polyunsaturated fatty acids and PE in brain membranes in a model of Alzheimer’s disease; this showed that MLR can modulate the biophysical properties of neuronal membranes [251]. Interestingly, β- and γ-secretases that process the amyloid precursor protein (APP) to form β-amyloid are embedded in the cell membrane, and their activities depend on the membrane lipid composition and structure. These facts suggest that the amyloidogenic processing of APP could be facilitated by alterations in the membrane lipid bilayer, and MLR could constitute an innovative approach to interdict this process [252]. Several studies carried out in cellula and in animal models of human Alzheimer’s disease (5XFAD mice) using the hydroxyalated polyunsaturated fatty acid DHAH and other fatty acids support this hypothesis and shed light on the molecular and cellular mechanisms involved in their therapeutic action. The changes in neuronal membrane lipids induced by DHAH cause dramatic inhibitions of amyloidogenic secretases activities, followed by reductions in amyloid loads, amyloid binding to membranes, and Tau protein phosphorylation. Moreover, 5XFAD mice treated with DHAH show normal cognitive scores and increased neurospore (neuronal stem cells) proliferation in the brain [251,253]. These studies indicate that MLR and related membrane lipid therapy (melitherapy) approaches can be used in the prevention and treatment of neurodegenerative processes associated with aging. Moreover, in homozygotic APOE-ε4 people (at highest risk for Alzheimer’s disease), diet supplementation with DHA is related to better neural and cerebrovascular neuroimaging (magnetic resonance imaging), consistent with a benefit against neurodegeneration [254].

Nutritional approaches to preventing neurodegeneration have also been applied to Parkinson’s disease. In a rat model of Parkinson’s disease, the omega-3 fatty acids DHA and DHAH induced important improvements in behavioral parameters associated with reductions in astrogliosis, microgliosis, and GFAP staining in brain striatum and substantia nigra [255]. These changes were markedly and significantly more pronounced in animals treated with DHAH, as this compound has a greater ability to incorporate into membrane GPL through its metabolite heneicosapentaenoic acid (21:5 n-3) [256]. Thus, lipid alterations and normalization can counter the development of pathophysiology of neurodegenerative diseases, such as Alzheimer’s and Parkinson’s diseases. Lipid composition influences membrane lipid bilayer structure, and the interactions of membrane proteins involved in normal and pathological protein functions and cell signaling, which justifies the use of MLR to prevent neurodegeneration [11,249].

## 11. MLR in Metabolic and Cardiovascular Diseases

As a common pre-disease condition found predominantly in the aging population, metabolic syndrome (MetSyn) is thought to be a precursor condition for several important age-related diseases, most such as type 2 diabetes (T2D). MetSyn is characterized by several interrelated blood disturbances of glucose and lipid homeostasis that are generally but not always found in obese, middle-aged individuals. These changes include: insulin resistance, changes in blood lipid profiles, abnormal glucose tolerance, hypertension, and vascular inflammation, as well as a background of multiple genetic abnormalities [257,258]. There are a number of major risk factors and blood markers routinely found in most MetSyn patients: (i) obesity or excess abdominal fat; (ii) elevated fasting plasma glucose levels; (iii) dyslipidemia, such as increased triacylglyerols, increased LDL, and reduced HDL in blood; (iv) elevated blood pressure; and (v) the presence of circulatory prothrombotic and proinflammatory molecules [257,258]. MetSyn (also called Syndrome X [259] or insulin-resistance syndrome [260]) is estimated to be present in over 40% of the over-60 age group in North America [260]. The same list of important risk factors in MetSyn patients are also found in NCD, such as hypertension, CVD, and other conditions [257,261,262].

Although there several blood markers and characteristics of MetSyn (listed above), the most important risk factors in the initiation of MetSyn are considered to be genetic abnormalities, obesity, and metabolic susceptibility (insulin resistance and other characteristics) [257,258,259,260,263]. Summarizing the factors that are involved in insulin resistance and MetSyn: (i) multiple genes; (ii) epigenetics, such as nutrition, low birth weight, and others; (iii) visceral obesity; (iv) body-mass index; (v) caloric and carbohydrate intake; (vi) sedentary lifestyle; (vii) age; (viii) ethnicity; (ix) gender; (x) menopausal status; (xi) alcohol consumption; (xii) inflammation; and (xiii) dysbiosis [264,265,266,267]. Thus, multiple risk factors, signs, symptoms, and abnormal laboratory test results can be used to generate a MetSyn diagnosis, but the relative merits of using the MetSyn diagnosis in clinical practice to direct patient management remains in development [263,264,267,268].

One of the lesser studied aspects of metabolic disorders are inflammation-associated changes involved in the generation of insulin resistance and MetSyn. These can result in the activation of NLRP3 inflammasomes by various mechanisms, including generation of mitochondrial DAMPs [269], lysosomal membrane disruption [270], and high fat diets [271]. The activation of inflammation complexes such as the NLRP3 inflammasome complex contributes to the development of visceral adiposity, endothelial dysfunction and insulin resistance, precursors to type 2 diabetes (T2D) [272]. The NLRP3 inflammasome complex may represent a signaling pathway that facilitates multiple organ metabolic damage [273]. The NLRP3 inflammasome also regulates the gastrointestinal microbiome and is activated by pathobionts and associated dysbiosis, which can affect host susceptibility to metabolic disease onset and progression [274]. Obesity, non-alcoholic fatty liver disease, and T2D, as well as changes in the integrity of the gastrointestinal barrier all affect lipid balance and homeostasis [275]. 

Lipids are critically involved in MetSyn and associated diseases, and defects in the capacity to utilize and metabolize lipids and glucose are especially important in insulin resistance and MetSyn [276]. Accumulations of DAG, triacylglycerol, and free FAs in non-adipose tissues correlate strongly with insulin resistance [277], and increases in released, free fatty acids may block insulin signal transduction and contribute to MetSyn [264]. Lipids, such as DAG, have been implicated in insulin resistance by activating isoforms of protein kinase C, which in turn can directly modulate insulin signaling by phosphorylating and inhibiting the insulin receptor tyrosine kinase and activating genes responsible for FA-induced inhibition of insulin activity [277]. Differences in gene expression in adipose tissue may be responsible for increasing the secretion of MetSyn-related factors, such as the pro-inflammatory cytokine TNFα and the tissue-specific protein adiponectin [278]. In muscle tissue, decreased oxidative capacity and fat accumulation may also induce skeletal muscle insulin resistance and contribute to the development of MetSyn, and eventually T2D [276]. Although MetSyn, T2D, and their associated diseases are known to be caused by multiple events, various studies point to mitochondrial dysfunction as a major component [265,268,269]. This has been shown in experiments where microarrays have been used to monitor gene expression in T2D. Such experiments revealed that several mitochondrial genes were down-regulated in T2D, supporting the notion that mitochondrial dysfunction is important in T2D [270]. Additionally, in muscle cells decreased ETC activity as well as decreased whole body anaerobic capacity are present in T2D patients, indicating involvement of mitochondrial dysfunction [271]. 

In T2D patients’ genetic polymorphisms have been found that are involved in FA oxidation and in factors that control transcriptional activities important in mitochondrial function [267]. Reduced fat oxidative capacities and increases in release of FA are found in MetSyn and T2D patients [279]. In obese, pre-diabetic, and diabetic patients, free FA levels are increased together with decreases in fat oxidative capacity, and this causes accumulation of FA and acylglyerols in pancreatic beta cells and other tissues. These changes correlate strongly with insulin resistance, MetSyn, and pre-diabetes [258,280]. In MetSyn, T2D, CVD, and renal diseases free FA can accumulate inside cells and in mitochondria, where they are prone to peroxidation, resulting in the formation of lipid peroxides. Lipid peroxides can be cytotoxic and result in free-radical damage to lipids, proteins, and DNA, especially in mitochondria. When the MIM is oxidatively damaged, MIM proton leakage and ETC function and mtDNA damage occurs. This type of damage can also result in activation of the NLRP3 inflammasome [281].

T2D is thought to develop from MetSyn due to persistent hyperglycemia, which in turn causes: (i) formation of advanced glycation end-products (AGEs), their accumulation and oxidative interactions with cell receptors; (ii) activation of various isoforms of protein kinase C; (iii) induction of the polyol pathway; and (iv) increased hexosamine pathway flux [282,283,284]. Most of these pathways are associated with elevated oxidative stress and over-production of ROS/RNS during hyperglycemia. This can result in MetSyn, and eventually the development of T2D [282]. Obesity cannot be underestimated in this process, as it results in hyperlipidemia, an increase in FA oxidation products that stimulate insulin secretion, and hyperinsulinemia. Hyperinsulinemia can, in turn, down-regulate insulin receptors and increase blood glucose levels [285]. Excess oxidative stress contributes to the progression of MetSyn to T2D by disrupting the ability of pancreatic beta cells to respond to elevated blood glucose levels [282,283]. The excess ROS/RNS eventually results in death of pancreatic beta cells by apoptosis, and this further reduces production of insulin [286].

Dietary supplements can prevent some of the damage to cellular and mitochondrial membranes seen during MetSyn development and potentially slow the process of progression to T2D. Such supplements are also important in preventing loss of ETC function seen in MetSyn and T2D [286]. Dietary use of various types of antioxidants or other supplements that increase free-radical scavenging systems have been employed to reduce or prevent the process of development of MetSyn and T2D [282,285,287]. In MetSyn, T2D, CVD, and diseases caused or promoted by continuing excess ROS/RNS, dietary supplementation with low molecular weight antioxidants, plus some replacements of accessory molecules have been employed. By adding enzyme cofactors such as zinc, manganese, copper, vanadium, chromium, and selenium ions necessary for antioxidant and some enzyme function plus certain vitamins with antioxidant properties and coenzymes (examples: C, E, A, CoQ_10_) such combinations can be used to maintain antioxidant levels and free-radical scavenging systems [267,282,285,288,289]. As discussed in previous sections of this review, supplementation with oral antioxidants, enzymes, vitamins, and other cofactors alone may not be sufficient to maintain cellular components and eliminate ROS/RNS damage. Thus, supplementation with antioxidants and needed cofactors alone cannot replace damaged cellular components, especially the GPL in cell and mitochondrial membranes [289,290]. Thus, as stated previously in this review, addition of MLR ingredients to the usual antioxidant supplements may be necessary for optimal maintenance of membrane health. 

MLR should also be useful in replacing membrane and blood components damaged by MetSyn, such as restoring unoxidized GPL in blood lipoproteins [1,289]. The administration of MLR phospholipids, along with changes in diet and lifestyle, can help to remove oxidized phospholipids and cholesterol from HDL and LDL [290]. Treating T2D patients with oral MLR supplements resulted in decreased serum triglyceride levels and reduced lipid peroxidation products compared to placebo controls [291]. There was also a significant reduction in the levels of acyl-hydroperoxides, Schiff’s bases, diene/triene conjugates, and MDA in patients taking oral MLR phospholipids [292]. Some studies found a significant reduction in blood sugar levels in patients with T2D given 1.2 g of oral MLR for 60 days compared to a control diet group [293]. Despite the evidence for a connection between excess oxidative stress in MetSyn, T2D, and associated diseases, an association between the intake of high concentrations of oral antioxidant nutrients and the prevention or delay of MetSyn progression to T2D and other diseases has not been realized [289,294,295]. Controlled clinical trials, for the most part containing single oral antioxidants, failed to show significant prevention benefits [296]. This should not be unexpected, as these complex phenomena involve multiple, complex mechanisms and pathways, and it is unlikely that a simple, singular approach could be effective.

An example of this complexity is the involvement of inflammation and vascular function in the development of metabolic diseases. For example, an important condition that usually precedes the development of MetSyn and T2D, hypertension, is directly related to vascular dysfunction, and this can be present with insulin resistance for many years before the development of T2D [297]. Hypertension is linked to insulin resistance, excess oxidative stress, mediated mainly by ROS/RNS, and changes in endothelial and smooth muscle cells. These are also known to be involved in vascular inflammation and initiation of apoptosis [298,299]. Chronic inflammatory damage to blood vessels due to lipid accumulation, inflammatory responses, endothelial cell death, and thrombosis, can eventually result in atherosclerosis and CVD. 

Atherosclerosis is characterized by a number of risk factors, including abnormalities in lipoproteins, increases in vascular acute phase response proteins, changes in vascular endothelial cell adhesion molecules, and the presence of certain inflammatory cytokines [299,300]. ROS/RNS plays an important physiological role in maintaining vascular integrity, but when present in excess, ROS/RNS can be involved in pathological processes. As mentioned above, excess production of ROS/RNS is associated with the development of MetSyn, T2D, hypertension, atherosclerosis, and CVD [289,299]. Endothelial and adipose dysfunction, along with insulin resistance, are thought to be among the most basic physiologic abnormalities that link MetSyn and CVD [300,301]. Vascular damage associated with excess oxidation, inflammation, and thrombosis is a primary event in the development of MetSyn, CVD, and other diseases [302]. Macrophages are also recruited to adipose tissue, and changes occur in adipose cells in parallel with changes in endothelial cells, such as induction of secreted adipokines and cytokines [301]. 

That MLR alone can modify or reverse the conditions described above and result in reversal of T2D, atherosclerosis, CVD, and other diseases remains extremely unlikely, if not completely impossible. However, the use of MLR phospholipids can change the composition and oxidation state of circulating lipoproteins and lipids [82,303]. Thus, this has the potential to modify pathologic processes and limit the progression of more lethal forms of metabolic diseases. In addition, MLR can also have other benefits. For example, the administration of MLR has resulted in the removal of cholesterol from serum lipoproteins and membranes [304]. In a 10-year study using rhesus monkeys fed high cholesterol diets, seven weeks of oral MLR with dietary lecithin lowered significantly total cholesterol, LDL cholesterol, and triglyceride blood levels [305]. Other studies have also shown that MLR supplements can reduce blood cholesterol, LDL-cholesterol, and triglycerides [258]. Another example is the MLR treatment of pigs that were previously fed a high cholesterol and coconut oil diet for 24 weeks as an experimental model of atherosclerosis. The pigs were then administered a MLR product (EPL) [306]. Without EPL, the serum levels of triglycerides, cholesterol free FAs, and beta-lipoproteins gradually increased with time, but with 8-weeks of EPL (up to 280 mg/kg body weight) there was a dose-related reduction in total lipids, cholesterol esters, free cholesterol, and triglycerides. At the highest EPL dose levels, there was a reduction in atherosclerotic plaques observed in the aortas and heart valves of the animals [306].

Interestingly, glycemia and blood pressure have been improved in elderly people who follow diets enriched in olive oil, and this occurs in connection with significant membrane lipid regulation and subsequent modulation of membrane PKC and G protein levels [307]. Olive oil is rich in unsaturated fatty acid, such as oleic acid (70–80%), which increases in content in membranes upon chronic supplementation with olive oil [307]. This and other studies showed that oleic acid regulates the interactions of PKC and G proteins with cell membranes, and these changes are associated with the health benefits seen in populations that have higher levels of olive oil consumption [307,308].

Excessive lipoprotein lipid peroxidation is one of the first changes associated with the development of MetSyn, and it is thought to also be important in the development of hypertension, T2D, atherosclerosis, and CVD. Thus, agents that reduce lipoprotein lipid oxidation may inhibit or attenuate the development of these diseases [289,309]. Or this could at least be a good marker for inhibition of progression to more lethal diseases. MLR has been shown to reduce lipid peroxidation in patients with ischemic heart disease. For example, patients with angina pectoris took an oral MLR supplement containing 1.8 g GPL per day for 3 weeks. At the end of treatment, there was a significant reduction in oxidized serum lipids, an increase in HDL cholesterol, and a reduction in erythrocyte hemolysis due to peroxidation [292]. Although these patients were not followed long enough to determine the long-term benefits of MLR, the prospect of long-term benefits of MLR needs to be examined in controlled clinical studies.

The use of MLR in clinical settings has demonstrated that blood levels of cholesterol, LDL-cholesterol, and triglycerides can be slowly reduced over time, and this should eventually result in health benefits. In long-term dialysis patients who are at risk for ischemic cardiovascular complications, patients tend to have high blood lipid values. In a double-blind, randomized study, groups of ten patients who had hyperlipidemia (serum cholesterol >260 mg/dL, LDL-cholesterol >180 mg/dL, and triglyerides >200 mg/dL) were given 2.7 g per day oral PC or placebo for 6-weeks [310]. The 6-week treatment was followed by a two-week wash-out period, and lipid parameters were determined at 2, 4, and 6 weeks of treatment. Two weeks after PC administration, there was a significant reduction in LDL-cholesterol of 32 mg/dL compared to the stable placebo controls. By 4 weeks, triglycerides were significantly decreased by 58.2 mg/dL, and by 6 weeks. there was a reduction in triglycerides of 43.3 mg/dL compared to the placebo group (at 4 weeks, −5.7 mg/dL and at 6 weeks, −11.4 mg/dL) [310]. Thus, MLR supplementation can alter blood lipid markers in hyperlipidemia to more favorable values. In a double-blind study of type II hyperlipidemia patients, participants received either three doses of oral polyenylphosphatidylcholine (0.9 g per day) or placebo, and their blood lipid levels determined over time. Total cholesterol and LDL-cholesterol were lowered significantly, and there was a downward trend in apoprotein B, triglycerides, and VLDL-cholesterol, and an upward trend in apoprotein A1 content compared to the placebo group [311]. As discussed above, MLR supplementation or treatment can be useful in altering markers of pathologic changes. Thus, adding MLR supplementation with alterations in diet and appropriate caloric restriction and macro- and micro-nutrient adjustments can reduce and replace oxidized phospholipids and cholesterol from HDL and LDL. The long-term consequences of such interventions need to be determined.

Thus, MLR supplementation has the potential to reverse some of the lipid changes that are important in MetSyn development and possibly prevent the formation of MetSyn-associated diseases and their progression to more lethal outcomes. It is interesting that the long-term use of MLR in the form of oral NTFactor^®^ and vitamins can significantly reduce blood markers for CVD risk. In one such study, CVD risk factors, such as homocyteine and fasting insulin levels, were brought to normal ranges found in controls within 6-months [128]. In a group of these patients with homocyteine levels above the threshold levels for high-risk for CVD, such as heart attack, hospitalization, and death, MLR with oral NTFactor^®^ resulted in a reduction in blood test results to the normal ‘safe’ ranges within 6 months [128]. This rather short-term study did not allow the necessary time to determine if the use of MLR and vitamin supplementation resulted in long-term health benefits, such as survival and avoidance of CVD-related hospitalization, but it was a promising start. Future studies should document whether MLR can impede or reverse the course of development and progression of MetSyn and its associated diseases and their long-term consequences.

## 12. MLR in Other Clinical Conditions

In addition to the many uses of MLR described in this review and elsewhere [1,2,3,4,5], and shown in Table 1 for NTFactor^®^ and NTFactor Lipids^®^, MLR has been used in laboratory animals and humans to treat other life-threatening conditions, such as extensive chronic and acute liver and kidney disease. These include: toxic liver and kidney damage caused by carbontetrachloride, alcohol, galactosamine, acetaminophen, tetracycline, solvents, detergents, thioacetamide, indomethacin, anesthetics, ionizing radiation, immune-mediated hepatitis, and others. MLR, mainly with EPL, was shown to reduce the toxic effects of these agents and promote organ repair and regeneration (reviewed by Gundermann [6]). In addition, there are other exposures and infections in humans where MLR has been useful, such as in the treatment of damage caused by fat embolism, non-steroidal anti-inflammatory drugs, liver-damaging anti-microbial drugs, lethal hepatic toxins, fatty liver conditions due to malnutrition, and infections such as hepatitis [6]. MLR could also be useful in alleviating some of the symptoms of COVID-19, especially in cases where symptoms persist long after there is no longer evidence for an active viral infection [312].

The treatment of viral hepatitis and liver cirrhosis using MLR GPL have been studied mainly in uncontrolled and in some controlled clinical trials [313,314,315]. Hepatitis patients treated with MLR using intravenous EPL reported improvements in dyspepsia, nausea, epigastric pain, fullness in the epigastrium, and other symptoms, as well as improvements in hepatomegaly and presence of ascites. Laboratory test results also showed improvements, and histological analysis of liver biopsies indicated some regeneration of hepatocytes [313]. In a controlled clinical study, patients with chronic hepatitis were treated for one year with intravenous EPL, resulting in significant reductions in hepatomegaly, liver enzymes, hepatic excretory capacity, and improvements in gamma-globulin and serum albumin levels compared to controls [314]. Patients with advanced liver cirrhosis were also treated with oral MLR phospholipids. After 3 months treatment, most blood tests improved and were found to be within the normal ranges. Symptoms also improved along with reductions in hepatomegaly and ascites [315]. In other studies, patients with moderately severe to severe cirrhosis caused by hepatitis B virus were treated with IV EPL for 3 months and compared to patients who only received a vitamin preparation. In the control group liver function remained similar to pre-treatment values, whereas in the EPL treatment group there were significant improvements in liver function in a majority of patients [316].

MLR has also been successfully used in chronic ambulatory peritoneal dialysis (CAPD). MLR GPL-treated CAPD patients showed increases in ultrafiltration with more electrolytes, creatinine, urea, and phospholipids released into the ascites fluid, which were removed by dialysis. MLR phospholipids were able to restore normal physiological conditions in CAPD patients with abnormal ultrafiltration rates. Symptoms improved and patients generally benefited significantly from the treatment [317].

MLR is safe to employ during pregnancy, and thus MLR has been used to treat pregnancy-associated health conditions. For example, during pregnancy, gestosis or toxemia can occur where patients present with hypertension, edema, and proteinuria. This is thought to be caused by chronic intravascular clotting and fibrin deposition in the uteroplacental bloodstream, which can affect uteroplacental perfusion and fetal development [318]. In the more severe cases, high levels of lipid peroxidation products have been found in the serum and erythrocyte membranes [319]. In these patients, IV or oral MLR phospholipids were administered twice daily at a dose of 500 mg per day during the last trimester of pregnancy. In the treated patients, edema subsided, liver and kidney function tests normalized, and other symptoms eventually subsided [320].

Finally, oral MLR with NTFactor^®^ or NTFactor Lipids^®^ has been used to reduce symptom severities associated with complex chronic infections such as *Mycoplasma* species and Lyme disease-associated multiple co-infections [123,321]. The use of MLR in these chronic bacterial infections was mainly administered as patient support dietary supplements, not as treatments for the infections themselves. It was proposed that MLR supplementation should be considered in such chronic infectious diseases as part of a normal patient support protocol.

## 13. MLR: Future Directions

MLR supplements containing GPL, antioxidants, and other ingredients are being used to repair and replace oxidatively-damaged membrane GPL in order to restore activities and functions of cellular membranes, organelles, cells, tissues, and organs, and generally improve quality of life in aging and chronic illnesses. Many cellular activities require energy supplied primarily by ATP produced in mitochondria, and damage to mitochondria, specifically mitochondrial membranes and ETC complexes, reduces overall cellular energy production [1,2,3]. MLR can be used to increase and restore MIM trans-membrane potential and recover mitochondrial membrane function and production of ATP. It can also be used to repair plasma and other membranes and also to remove damaged membrane components and other hydrophobic toxic molecules. Clinical trials have shown the usefulness of MLR supplements in reducing symptoms associated with loss of mitochondrial and other cellular functions and improving the quality of life in patients with a variety of chronic illnesses and normal age-related loss of function [1,2,3]. Recent efforts have focused on the effects of MLR supplements on reducing pain, gastrointestinal, and other symptoms, as well as age-related functional loss. In these more recent studies, the recommended daily doses of MLR supplements, such as NTFactor Lipids, have generally increased in amounts (Table 1). In addition to the use of MLR in slowing or reversing functional loss in advanced age, MLR supplements can also be useful at the earliest stage of life, for example, in enhancing sperm motility, increasing fertility, and reducing the effects of oxidative stress on sperm and ova membranes and protecting gametes during cryogenic storage [33,322]. 

An important new use of MLR GPL is the potential of MLR to remove damaged lipids and toxins by an exchange process that is driven by bulk flow or mass action principals. This process and its dependence on mass action have the potential to reverse the presence of damaged GPL in organs, tissues, cells, and cellular membranes by exchange and transport to the lymph and blood circulation and eventual deposition in the gastrointestinal system and removal in stool [32]. The exchange and replacement of GPL in cellular membranes releases the damaged GPL, and after partitioning in lipid globules, lipid vesicles, chylomicrons, and lipoproteins, and exportation from cells into the circulation, the partitioned GPL can be transported by lipoproteins, blood cells, and other carriers and deposited in the gastrointestinal system. Eventually, the damaged GPL can be eliminated by excretion in stool. This mass action process explains the usefulness of MLR in the slow, bulk flow removal of molecules such as oxidized GPL, oxidized cholesterol, and oxidized fragments of other hydrophobic molecules as well as toxic, fat-soluble chemicals from hydrophobic cellular stores and their elimination.

The use of MLR phospholipids for repairing and replacing membrane GPL and returning oxidized GPL and cholesterol to the gastrointestinal system appears to be dose-dependent and requires some time to accomplish. Cross-over clinical trials have suggested that the use of MLR supplements needs to be continued as a long-term strategy for support of chronic diseases (or age-related membrane damage), as temporary changes due to MLR supplementation can be slowly reversed [1,2,3]. Even in cases of hydrophobic toxic insult or removal of hydrophobic toxins that have been partitioned into membranes and other hydrophobic centers, MLR administration must be a slow, long-term process, as mobilizing and removal of toxic molecules too quickly can result in symptomatic effects. Thus, the removal of deeply embedded toxic molecules must occur at a slow, steady pace that could require many months or even years of MLR supplementation to accomplish. However, some uses of MLR may not take more than several minutes, such as the ex vivo treatment of sperm or ova.

Some benefits of MLR may not be related to removal of GPL, but instead are likely to be related instead to simple lipid substitution in cellular membranes, such as modulation of ion channels in membranes by MLR supplements to reduce pain and other symptoms. For example, exchanged GPL can modulate the functions of many, if not most cell surface ion channels, transporters, and receptors [323,324,325,326,327,328]. Among numerous possible membrane channels, voltage-gated calcium channels, as well as intracellular calcium channels are particularly important in nerve cells in maintaining trans-membrane potentials and homeostasis [323,327]. Such effects of GPL may explain the effects of MLR on pain and other symptoms [214,215].

MLR may also be useful as an adjunct to more traditional drug approaches, either to enhance cell and tissue absorption or increase circulatory transport of drugs, modify cellular energy requirements, or change drug and messenger membrane interactions [1,2,3,4]. The design of new MLR formulations, and especially the use of MLR phospholipids in combination with other health supplements, will prove to be extremely useful for future specific health uses.

## Figures and Tables

**Table 1:** Current and potential uses of oral MLR supplements and revised dose levels ^a^.

Use	Subjects/Patients	Age	MLR Lipid	NTFL Dose ^b^	NTFL Dose ^c^	Example
		Group	Supplement	Range (g/day)	Range (g/day)	Reference
				(Original)	(Revised)	
General health	Aged	senior	NTFactor/L ^d^	2	2–3	Nicolson et al. [3]
Fatigue	Aged	senior	NTFactor/L	3	4	Agadjanyan et al. [145]
Fatigue	CFS/ME	adult/teen	NTFactor/L	2–4	4	Nicolson & Ellithorpe [203]
Fatigue	CFS/ME	adult	ATP Fuel	4	4	Nicolson et al. [123]
Inflammation	Chronic fatigue	adult	ATP360	0.4	N/A ^e^	Hamilton & Jensen [206]
Fatigue	Fibromyalgia	adult	NTFactor/L	3–4	4	Nicolson et al. [215]
Fatigue	Menopause	adult	NTFactor/L	1.2	3	Hirose et al. [174]
Weight loss	Obesity, fatigue ^d^	adult	NTFactor	2	3–4	Ellithrope et al. [245]
Brain health	Neurodegen. dis.	adult	NTFactor/L	3–4	4	Nicolson et al. [175]
CD health	CD risk/CD dis.	adult	NTFactor/L	2–4	4	Ellithorpe et al. [128]
Metabolic health	MetSyn/diabetes	adult	NTFactor/L	2–4	4	Nicolson [289]
Metabolic health	Diabetes	adult	ATP Fuel	4	4	Nicolson et al. [123]
Neurobehavior	Autism Spectrum dis.	child	NTFactor/L	1–2	1–3	Nicolson et al. [175]
Infections	Lyme/mycoplasma	adult	ATP Fuel	4	4	Nicolson et al. [321]
Fertility	Fertility Diseases	adult	NTFactor/L	2–3	4	Ferreira et al. [33]
Fatigue	Cancer	adult	NTFactor/L	2–3	4	Nicolson & Conklin [197]
Anemia	Anemia	adult	NTFactor/L	1–2	4	Nicolson et al. [123]
Injury	Spinal injury	adult	NTFactor/L	1–2	4	Ellithorpe et al. [123]
Autoimmune	Rheumatoid arthritis	adult	ATP Fuel	3	4	Nicolson et al. [123]
General health	Pregnancy	adult	NTFactor/L	1–2	2–3	Ellithorpe et al. [205]
Chemical detox	GW Illnesses	adult	NTFactor/L	>4	>6	Nicolson & Breeding [214]

^a^ Modified from Nicolson et al. [1]. ^b^ Dose range in grams per day based on NTFactor Lipids^®^. ^c^ Revised dose range in grams per day based on NTFactor Lipids^®^. ^d^ NTFactor^®^ or NTFactor Lipids^®^. ^e^ Not Available.

## Data Availability

Not applicable.

## Abbreviations

ABR	auditory brainstem responses
AD	Alzheimers disease
AGEs	advanced glycation end products
CAPD	chronic ambulatory peritoneal dialysis
CFS	chronic fatigue syndrome
CL	cardiolipin
CoQ10	coenzyme Q10
CVD	cardiovascular disease
DAG	diacylglycerol
DAMPs	damage associated molecular patterns
DHA	docosahexaenoic acid
EPA	eicosapentaenoic acid
EPL	essential phospholipids
ETC	electron transport chain
FA	fatty acid
FDA	US Federal Drug Administration
GPL	glycerolphospholipids
GRAS	generally recognized as safe
HDL	high density lipoproteins; HNE 4-hydroxynonenal
IL	interleukin
MDA	malondialdehyde
MetSyn	metabolic syndrome
MIM	mitochondrial inner membrane
MLR	membrane lipid replacement
mPTP	mitochondrial permeability transition pores
mtDNA	mitochondrial DNA
NCD	non-communicable diseases
NF-κB	nuclear factor kappa B
PC	phosphatidylcholine
PE	phosphatidylethanolamine
PG	phosphatidylglycerol
PI	phosphatidylinositol
PS	phosphatidylserine
RNS	reactive nitrogen species
ROS	reactive oxygen species
TNFα	tumor necrosis factor alpha
T2D	type 2 diabetes
